# The Correlation Between Fractional Exhaled Nitric Oxide (FeNO), Blood Eosinophil Count, Immunoglobulin E Levels, and Spirometric Values in Patients With Asthma

**DOI:** 10.7759/cureus.35289

**Published:** 2023-02-22

**Authors:** Mohammed O Al Ghobain, Abdullah S Alsubaie, Walaa A Aljumah, Fahad M Alrumayh, Khalid F Aldawsari, Asma M Alqahtani, Sultan N Alotaibi

**Affiliations:** 1 Medicine, King Abdulaziz Medical City, Riyadh, SAU; 2 Medicine, King Saud bin Abdulaziz for Health Sciences, Riyadh, SAU; 3 Medicine, University of Jeddah, Jeddah, SAU; 4 Medicine, Qassim University, Qassim, SAU; 5 Medicine, Prince Sattam Bin Abdulaziz University, Kharj, SAU

**Keywords:** asthma, spirometric values, immunoglobulin e levels, blood eosinophil count, fractional exhaled nitric oxide (feno)

## Abstract

Background and objective: In patients with asthma, fractional exhaled nitric oxide (FeNO) is a biomarker for eosinophilic airway inflammation. However, the correlation with the blood eosinophil count, immunoglobulin E (IgE), and spirometric values is not well established. We aimed to investigate the correlation between FeNO, blood eosinophils, IgE, and spirometric values in asthmatic patients.

Methods: Data were extracted from the electronic medical records of adult asthmatic patients, and included the FeNO, blood eosinophil count, IgE, and spirometric values. The correlation between the markers was investigated using receiver operating characteristics (ROC) and the area under the curve (AUC).

Results: A total of 135 adult patients (30% male) were analyzed. The mean FeNO was 36 ppb and almost half (48%) had a FeNO > 25 ppb. The mean blood eosinophil count was 377 cells/mm^3^, and 42% had a blood eosinophil count > 300 cells/mm^3^. There was a significant positive correlation between FeNO and the blood eosinophil count (r = 0.42, sensitivity 63%, specificity 77%, AUC 72%, p < 0.0001) as well as with the IgE (r = 0.35, sensitivity 78%, specificity 44%, AUC 68%, p < 0.0007). There was significant negative correlation between FeNO and FEV1% (r = -0.18, sensitivity 35%, specificity 85%, AUC 67%, p = 0.027).

Conclusion: The blood eosinophil count, IgE, and spirometric values were correlated with the severity of the eosinophilic airway inflammation (high FeNO). Asthmatic patients with a higher FeNO level had a higher eosinophil count, higher IgE levels, and lower FEV1 values.

## Introduction

Asthma is a heterogeneous disease characterized by chronic airway inflammation [1]. It is defined by a history of respiratory symptoms, including wheezing, shortness of breath, chest tightness, and cough that vary over time and in intensity, as well as a variable expiratory airflow limitation [1]. The diagnosis of asthma is based on symptoms and a physical examination in combination with pulmonary function tests (spirometry) to evaluate airway hyper-responsiveness and to detect reversible obstruction [1].

Because asthma is a heterogeneous and complex disease, an assessment based on symptoms and spirometry alone does not accurately represent the underlying airway inflammation, the patient characteristics or profile (phenotype), or the underlying mechanism of airway inflammation (endotype) [2]. We aimed to establish a tool to measure airway inflammation by measuring specific inflammatory biomarkers, including fractional exhaled nitric oxide (FeNO), blood eosinophil count, and serum immunoglobulin E (IgE) [2]. Measuring such biomarkers can assist in the diagnosis and prognosis, guide treatment options and predict and monitor the response to therapy [2] and support the achievement of the goal of treatment. In asthma, the goal is symptom control, the maintenance of normal activity levels, and a reduction in the future risk of exacerbations and fixed airflow limitations [1].

The FeNO measurement is an easy and non-invasive test. A FeNO concentration greater than 25 parts per billion (ppb) in adults is a marker of eosinophilic airway inflammation and predicts the likelihood of corticosteroid responsiveness [3,4]. An elevated FeNO is considered a risk factor for uncontrolled asthma and future exacerbations [5]. The peripheral blood eosinophil count is also considered a biomarker for airway inflammation; a high level is associated with future risk of asthma exacerbations and developing a fixed airflow limitation [2,6]. Serum IgE, produced by the plasma cells, is considered another biomarker for airway inflammation but the significance of IgE as a biomarker for eosinophilic asthma has not been proven and it is currently not recommended [6].

Several studies attempted to establish a correlation between the blood eosinophil count and FeNO, but with contrary findings. A study reported no relationship between FeNO and the blood eosinophil count in uncontrolled asthma [7]; however, another study showed that FeNO was significantly correlated with the blood eosinophil count in uncontrolled childhood asthma [8] and in uncontrolled adult asthmatic patients [9]. It is demonstrated that the prevalence of uncontrolled asthma and impaired lung function increased with an increase in the blood eosinophil count and the FeNO level, compared with asthma patients with an increased blood eosinophil count and normal FeNO [10]. A high IgE, FeNO, and absolute eosinophil count were found in patients with atopic asthma, compared with patients with non-atopic asthma [11]. Regarding the relationship between the blood eosinophil count and the spirometric values, the eosinophilic phenotype of asthma is associated with asthma severity and poor symptom control, but not associated with impaired lung function [12]. In contrast, a higher eosinophil count was associated with lower FEV1% predicted values for both pre- and post-bronchodilator spirometry and a faster decline in spirometric values [13].

As most of the studies investigating the correlation between the biomarkers in asthma reported conflicting results with variable conclusions, we aimed to investigate the correlation between FeNO, the blood eosinophil count, serum IgE level, and spirometric values in patients with asthma.

## Materials and methods

This was a retrospective analytical study, conducted at the Pulmonary clinic at King Abdulaziz Medical City in Riyadh, Saudi Arabia. Both genders, 15 years and older, with a diagnosis of asthma, and FeNO, spirometry tests, and the blood eosinophil count results available in the electronic medical records system, from June 2019 to June 2022, were included in the study. The sampling technique was non-probability purposive sampling. Smokers and patients with chronic pulmonary disease or significant co-morbidity which can affect the value of FeNO (malignancy, hematological disease, vasculitis, organ failure, respiratory failure, stroke) were excluded.

The data collected consisted of the baseline characteristics, including age, gender, body mass index (BMI), and duration of asthma diagnosis as well as an asthma control test (ACT). The other variables were the FeNO, eosinophil count, IgE, forced vital capacity (FVC), forced expiratory volume in the first second (FEV1), and the ratio of FEV1/FVC as well as bronchodilator reversibility. In this study, the level of FeNO was categorized into two groups: high FeNO (FeNO ≥ 25 ppb) and low FeNO (FeNO < 25 ppb). The level of the blood eosinophil count was categorized as a high blood eosinophil count (≥ 300 cell/mm^3^) and low blood eosinophil count (< 300 cell/mm^3^). Asthma control was assessed using the Asthma Control Test (ACT > 20 was considered as controlled asthma and < 20 uncontrolled). The patients were subdivided into impaired or normal lung function values (FEV1 < 75% or ≥75%, FVC < 75% or ≥75% of predicted value, or FEV1/FVC < 75%). Bronchodilator reversibility was defined as >200 mL and > 12% post-bronchodilator. The study protocol was approved by the Institutional Review Board of King Abdullah International Medical Research Center.

All statistical analyses were conducted using SPSS Statistics version 23 for Windows. The demographic data were summarized in frequency tables. The categorical values, expressed as frequency and percentage, were analyzed using a Chi-square test. The continuous or ordinal values were summarized as a mean ± SD and analyzed with the non-parametric Mann-Whitney U test. The correlation between the markers was investigated using receiver operating characteristics (ROC) and the area under the curve (AUC). A p-value ≤ 0.05 was considered statistically significant.

## Results

A total of 135 patients with asthma met the inclusion criteria. The baseline characteristics of the patients are displayed in Table 1. The mean age was 40 years (30% male). The majority (82%) of the patients had an asthma duration of more than five years, and 64% had uncontrolled asthma (ACT<20). The mean FeNO was 36 ppb, with a range of between 2 and 226 ppb. Almost half (48%, n=65) were in the high FeNO category. The mean absolute eosinophil count was 377 cells/mm^3^ and the mean percentage was 4.6%. Less than half (42%) had an absolute eosinophil count > 300 cells/mm3. The mean IgE level was 311 KU/L, and the majority (68%) had an IgE > 100 KU/L. The mean FEV1%, FVC%, and FEV1/FVC ratio were 85%, 91%, and 78% respectively. Of the group with reduced spirometry values (ratio less than 75%), the mean FEV1%, FVC%, and FEV1/FVC ratio were 56%, 63%, and 64% respectively. The mean bronchodilator reversibility value was 192 mL, and 48% had a bronchodilator reversibility response (> 200 mL).

**Table 1:** Baseline characteristics of the sample ACT = asthma control test, FEV1 % = forced vital capacity in first second, FVC=forced vital capacity.

Categories	
Age (year)	
mean (SD) range	40 (13) (15-80)
15-30 : n %	36 (26.7%)
31-50 : n %	57 (42.2%)
>50 : n %	42 (31.1%)
Gender:	
Male: n ( %)	41 (30%)
Female: n ( %)	94 (70%)
Body mass index kg/m	
mean (SD) range	30 (17-46)
<18 n (%)	1 (0.7%)
18-25 n (%)	25 (18.5%)
26-30 n (%)	43 (31.9%)
>30 n(%)	66 (48.9%)
ACT number of available data: 97	
≥ 20 (controlled) number %	35 ( 36%)
< 20 (uncontrolled) number %	62 ( 64%)
Asthma duration (years) number of available data: 120	
≥ 5 years n (%)	98 (82%)
< 5 years n (%)	22 (18%)
FeNO level	
Total patients: FeNO level, ppb mean (SD) range	36 (37) ( 2-226)
FeNO ≥ 25 ppb number(%), mean (SD) range	65 (48%) ( 62 ) (39) (25-226 )
FeNO < 25 ppb number (%) , mean (SD) rangE	70 (52%) (12) (5) (5-24)
Eosinophil count, cell/mm	
Total patients : mean (SD) range	377 (440) ( 0-2700)
Eosinophil ≥ 300 cell/mm^3 ^: number(%) mean (SD) range	57(42%) ( 711) (508) ( 300-2700 )
Eosinophil < 300 cell/mm^3^ : number(%) mean (SD) rangE	78 ( 58%) ( 133) ( +-81) ( 0-290)
Eosinophil %	
mean (SD) range	4.6% (4.3%) (0-27%)
≥ 3% : number (%), mean (SD) range	69 (51%) 7.7% (+-4.6%) (3-27%)
< 3% : number (%), mean (SD) range	66 (49%) 1.5% (+-0.9%) (0-2.9% )
IgE level ku/L	
number of available data : 91, mean (SD) range	311 ( 412 ) ( 8-2646 )
≥ 100 ku/L number(%), mean (SD) range	61(68% ) ( 437 ) (450) ( 105-2646 )
< 100 ku/l number(%), mean (SD) rangE	30 ( 32%) ( 46) (25) ( 8-97)
FEV1 %	
FEV1 % predicted, mean (SD)	85% (22%)
≥ 75% number(%), mean (SD)	101 (75%) ( 95% ) (14% )
< 75% number(%), mean (SD)	34 (25% )( 56% ) (14%)
FVC %	
FVC % predicted, mean (SD)	91% (17%)
≥ 75% number(%), mean (SD)	113 (84%) (97%) (12%)
< 75% number(%), mean (SD)	22(16%) (63%) (9%)
FEV1/FVC%	
FEV1/FVC% predicted, mean (SD)	78% (10)
≥ 75% number(%), mean (SD)	98(72%) ( 83% ) ( 5% )
< 75% number(%), mean (SD)	37(28%) ( 64%) (8%)
Bronchodilator reversibility	
Bronchodilator reversibility, number of available data : 81 , mean (SD)	192 ( 174 ) ( -230-800 )
≥ 200 mL number(%), mean (SD)	38 ( 48% ) 245 (172) ( 200-800)
< 200 mL number(%), mean (SD)	46 (58%) 89 (71 ) (-230-190)
Bronchodilator reversibility of >200 mL and >12%	
mean (SD)	60
≥ 200 mL and 12%, mean (SD) range	(406) (190) (210-800)
<200 mL and 12%, mean (SD) range	(84) (271) (-230-190)
Bronchodilator reversibility %	
Bronchodilator reversibility %, mean (SD)	(9.1%) (8.7%) ( -7-50%)
≥12% , mean (SD)	(19%) (9%) (12-50%)
<12%, mean (SD)	(4.7%) (3.5%) (-7-11%)
Sample (n= 135)	

There was no significant difference between the high and low FeNO groups in terms of age, gender, BMI, ACT and the duration of the disease. There were significant differences between the two groups and the eosinophil count and IgE. The patients in the high FeNO group also had a higher eosinophil count and IgE in comparison to the patients in a low FeNO group (p < 0.0001 and < 0.0028, respectively). Regarding the spirometric values, there was a significant difference between the two groups as the patients in the high FeNO group had a lower FEV1 (mean 78%), FEV1/FVC ratio (mean 74%) and bronchodilator reversibility compared to the patients in the low FeNO group (p < 0.046, < 0.016 and < 0.044, respectively) (Table 2).

**Table 2:** Data according to a level of FENO in the sample ACT = asthma control test, FEV1 % = forced vital capacity in first second, FVC=forced vital capacityACT = asthma control test, FEV1 % = forced vital capacity in first second, FVC=forced vital capacity.

	FENO ≥ 25 PPB (N=65 )	FENO < 25 PPB (N = 70 )	P-VALUE
Age (year) mean (SD)	39 (13)	42 (14)	0.3549
Male : n (%)	20 (30)	21 (30)	0.9200
Female : n (%)	45 (70)	49 (70)	0.9200
Body mass index unit, mean (SD)	29 (5)	31(6)	0.1420
ACT ≥ 20 (controlled) number %	15 (23)	20 (28.6)	0.2000
ACT < 20 (uncontrolled) number %	35 (54)	27 (38.6)	
Asthma duration (years) ≥ 5 years number %	45 (69.2)	53 (75.7)	0.2600
Asthma duration (years) < 5 years number %	13 (20)	9 (12.9)	
Eosinophil count, cell/mm^3^ , mean (SD)	537 (546)	228(231)	<0.0001
Eosinophil ≥ 300 cell/mm^3 ^, mean (SD)	769 (568)	562(267)	0.2553
Eosinophil < 300 cell/mm^3 ^ mean (SD)	141(95)	129(75)	0.5621
Eosinophil, % mean (SD)	6.3(5.5)	3.1(2.7)	<0.0001
Eosinophil % ≥ 3% , mean (SD)	8.5(5.2)	6.1(2.6)	0.0821
Eosinophil % < 3% , mean (SD)	1.4(1.1)	1.5( 0.8)	0.5815
IgE level, mean (SD)	391(443)	223(361)	0.0028
IgE level ≥ 100 ku/L, mean (SD)	483(450)	365(436)	0.0777
IgE level < 100 ku/L, mean (SD)	52(28)	43(23)	0.4085
FEV1 % , mean (SD)	78(20)	92(21)	0.0662
FEV1 % ≥ 75% ,mean (SD)	90(12)	99(15)	0.0009
FEV1 % < 75% , mean (SD)	65(14)	57(14)	0.6963
FVC % , mean (SD)	88(15)	94(18)	0.2600
FVC % ≥ 75% , mean (SD)	94(10)	99(13)	0.0611
FVC % < 75% , mean (SD)	64(9)	63(10)	0.8015
FEV1/FVC% , mean (SD)	74(10)	82(9)	0.0169
FEV1/FVC% ≥ 75% , mean (SD)	81(3)	85(5)	< .0001>
FEV1/FVC%< 75% , mean (SD)	63(8)	67(7)	0.1183
Bronchodilator reversibility ml, mean (SD)	224(179)	152(162)	0.0447
Bronchodilator reversibility ml ≥ 200 mL, mean (SD)	353(+-175)	331(+-173)	0.9473
Bronchodilator reversibility ml < 200 mL, mean (SD)	102(+-58)	78(+-80)	0.2400
Bronchodilator reversibility %, mean (SD)	10.8(+-9.8)	7(+-6.8)	0.0748
Bronchodilator reversibility % ≥ 12%, mean (SD)	21(+-10)	15(+-6)	0.0531
Bronchodilator reversibility % < 12%, mean (SD)	5.5(+-3)	3.9(+-3.8)	0.1089
Bronchodilator reversibility of >200 mL and >12%, mean (SD)	418(186)	381(212)	0.6928

**Figure 1:**
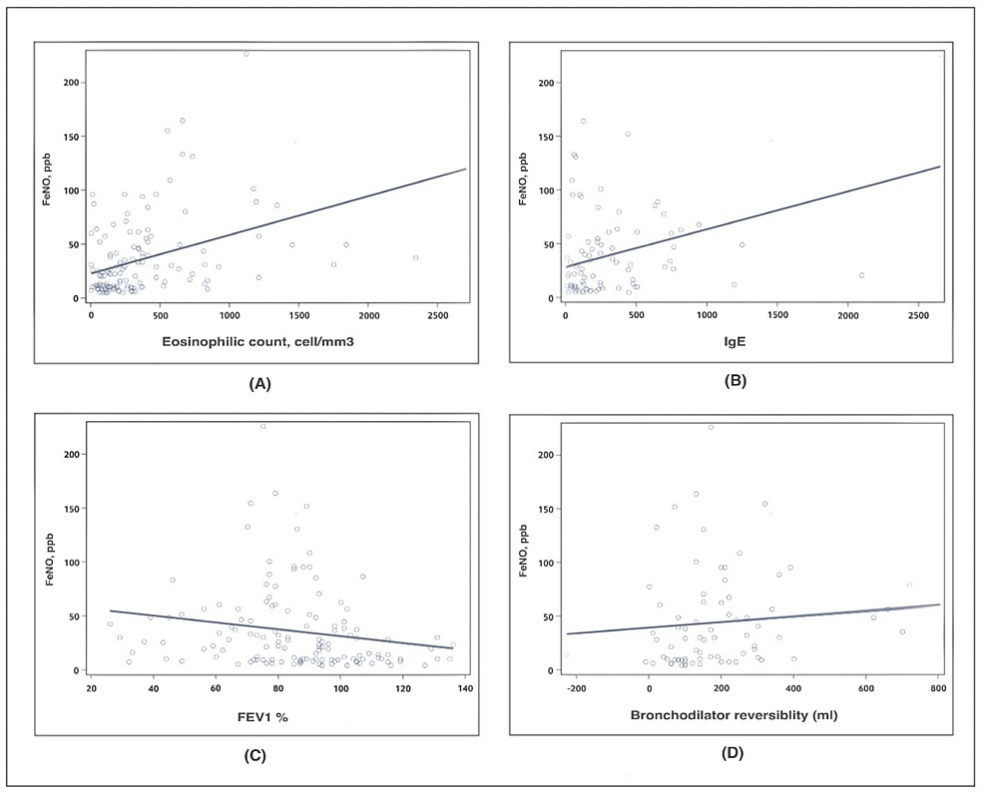
Scatter plots of (a) the correlation between FeNO and blood eosinophils (r = 0.42, p < 0.0007), (b) the correlation between FeNO and total IgE (r = 0.35, p < 0.0007), (c) the correlation between FeNO and FEV1% (r = -0.18, p = 0.0278), and (d) the correlation between FeNO and bronchodilator reversibility (r = 0.09, p = 0.044). FeNO: Fractional exhaled nitric oxide, IgE: immunoglobulin E, FEV1: Forced expiratory volume.

**Figure 2:**
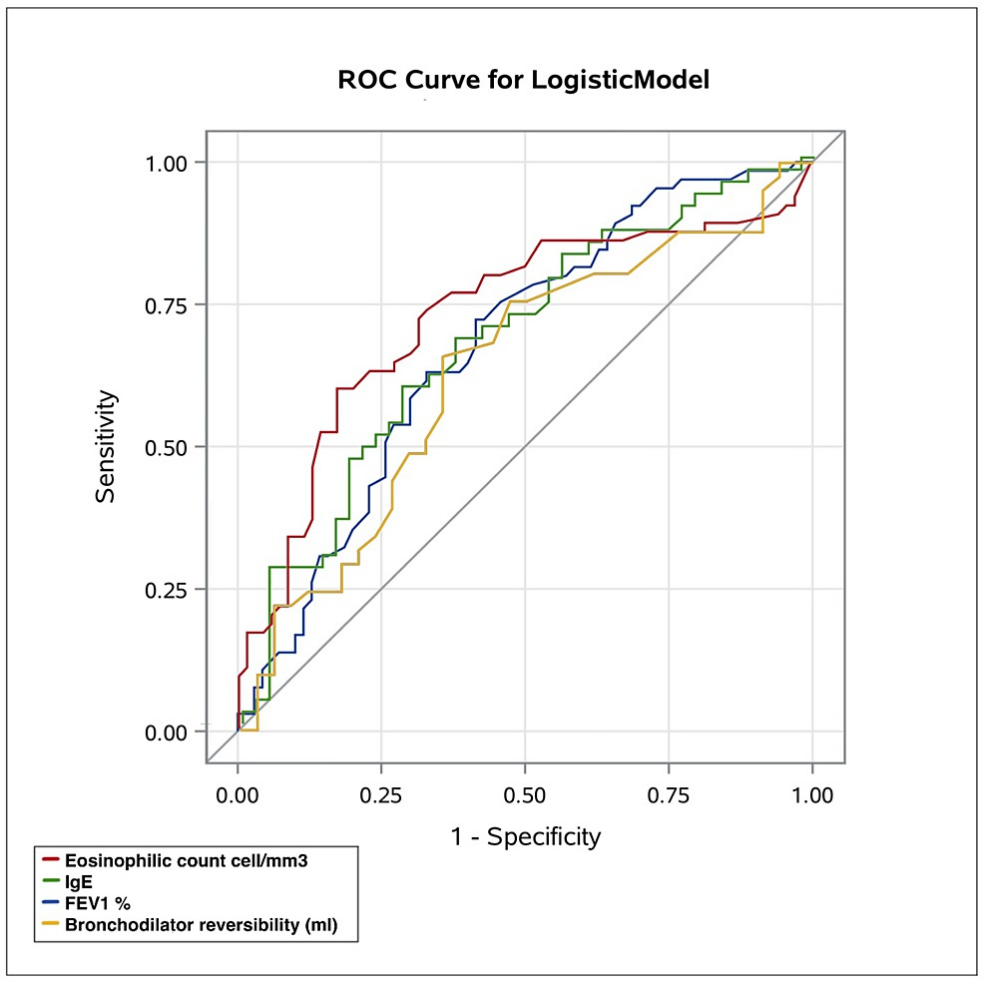
The receiver-operating characteristic curve analysis of sensitivity and specificity of each marker for identification of FeNO> 25 ppb (a) Blood eosinophils, cut-off point was: 300 cells/mm^3^, sensitivity 63%, specificity 77%, AUC:72%, p < 0.0001. (b) Total IgE, cut-off point was: 100 ku/L, sensitivity 78%, specificity 44%, AUC 68%, p < 0.0007. (c) FEV1, cut-off point was 75%, sensitivity 35%, specificity 85%, AUC 67%, p = 0.0278. (d) Bronchodilator reversibility, cut-off point was 200 mL, sensitivity 48%, specificity 70%, AUC:0.635, p = 0.4053. ROC: The receiver-operating characteristic curve, FeNO: Fractional exhaled nitric oxide, IgE: immunoglobulin E, FEV1: Forced expiratory volume.

There was a significant positive correlation between the FeNO levels and the blood eosinophil count (r = 0.42, p < 0.0001), and between the FeNO levels and the IgE levels (r = 0.35, p < 0.0007) (Figure 1). The ROC curve analysis of the sensitivity and specificity of the blood eosinophil count for the identification of the FeNO ≥ 25 ppb at an optimal cutoff point of 300 cell/mm3, had a sensitivity 63%, specificity 77%, AUC 72% (p < 0.0001). The ROC curve analysis of the sensitivity and specificity of the blood IgE for the identification of FeNO ≥ 25 ppb at an optimal cutoff point, of 100 KU/L, had a sensitivity of 78%, specificity 44%, AUC 68%, p < 0.0007 (Figure 2).

In terms of the spirometric values, there was a significant negative correlation between the FeNO level and the FEV1% (r = - 0.18, p = 0.027), and a weakly positive significant correlation between the FeNO level and bronchodilator reversibility (r = 0.09, p = 0.044) (Figure 1). The ROC curve analysis of the sensitivity and specificity of FEV1% for the identification of FeNO ≥ 25 ppb at an optimal cutoff point (75%) had a sensitivity of 35%, specificity 85%, AUC = 67%, p = 0.027. The ROC curve analysis of the sensitivity and specificity of the bronchodilator reversibility for the identification of FeNO ≥ 25 ppb at an optimal cutoff point (200 mL) had a sensitivity of 48%, specificity 70%, AUC=63% p = 0.4053 (Figure 2).

## Discussion

In this cohort of asthmatic patients, the FeNO was positively correlated with the blood eosinophil count (r = 0.42, p < 0.0001). In addition, a positive correlation was found between FeNO and serum IgE (r = 0.35, p < 0.0007). However, a significant but negative correlation existed between FeNO and FEV1 (r = -0.18, p = 0.027), and a significant correlation between FeNO and bronchodilator reversibility (r = 0.09, p = 0.004). There was no significant correlation between FeNO and asthma control (p = 0.12). Patients in the high FeNO group had a higher eosinophil count, higher IgE levels, and lower spirometric values.

As inflammation is the main pathognomonic feature of asthma, measurement of its biomarkers is important to establish the diagnosis of asthma, assess the severity of the disease and monitor the response to therapy. FENO is a non-invasive, inexpensive, and easy-to-perform test used with the blood eosinophil count as a biomarker for eosinophilic airway inflammation, and the level correlates well with type 2 asthma and the severity of inflammation. The current findings confirm the correlation between FeNO and the blood eosinophil count as an indication of the severity of eosinophilic airway inflammation, also supported in the literature. FeNO was significantly correlated with the blood eosinophil count in uncontrolled childhood asthma 8 as well as in uncontrolled adult asthmatic patients [9]. A study done in Saudi Arabia reported a positive correlation between FeNO and the blood eosinophil count. However, the sample size was small and they did not study the correlation between FeNO and bronchial reversibility [14].

The current findings demonstrated a significant and positive correlation between FeNO and the serum IgE level. Patients in the higher FeNO group had higher IgE levels. Strunk et al. [15] reported similar results, indicating that in asthma patients, the FeNO levels were correlated with the blood eosinophil count and IgE level. The serum IgE appears to be the least supportive of all the available biomarkers to identify eosinophilic asthma [6]. However, based on our results, the serum IgE level may reflect severe underlying airway inflammation as it was significantly correlated with higher FeNO levels.

However, we found a significant negative correlation between FeNO and FEV1% predicted. The spirometry measurements are lower with increasing inflammation (increasing FeNO level). A study indicating no correlation between FENO and the spirometric values considered them as different physiological parameters for diagnosing asthma [16]. FeNO and the blood eosinophil count in relation to the FEV1 were evaluated in 410 patients treated for asthma. The patients with an elevated FeNO and an elevated blood eosinophil count had impaired lung function (lower FEV1%) compared to the patients with a normal FeNO and blood eosinophil count [10]. He et al. [17] reported a negative correlation between FEV1 and FeNO in asthma patients.

Interestingly, the current findings found no significant correlation between FeNO and asthma control (P-value = 0.12). A patient may have a high FeNO (airway inflammation) but controlled asthma (ACT>20) and vice versa. This can be explained by the definition of severe eosinophilic asthma, which is not the same definition as uncontrolled asthma [1]. Another possible explanation is that in the current cohort, the proportion of patients with controlled asthma was small as only a third had controlled asthma (ACT > 20). However, Habib et al. [18] reported contrary findings, as they reported a significant negative correlation between FENO and the ACT score. The FENO values were significantly higher in the patients with uncontrolled asthma (lower ACT score). Malinovschi et al. [10] investigated FeNO and the blood eosinophil count in relation to asthma control. The patients with an elevated FeNO and blood eosinophil count had a higher prevalence of uncontrolled asthma (ACT <20) than the patients with only an increased blood eosinophil count. Lima-Matos et al. [12] demonstrated that the patients with the eosinophilic phenotype of asthma had severe asthma with increased odds of poor symptom control.

The current study had several advantages. These included a large sample size, exclusion of several confounding factors such as smoking and chronic lung diseases other than asthma, using optimal cut-off points of 25 ppb for FeNO as recommended by the American Thoracic Society, and finally, an optimal cut-off point of 300 cell/mm3 for the eosinophil count, as recommended by GINA [1].

A limitation of the study is the retrospective analysis of data from a single pulmonary function laboratory. In addition, some of the variables have been measured on separate occasions which were minimized by establishing a three-month data collection period between all the tests. Another limitation is not investigating the correlation between the FeNO and the risk of exacerbation. Choosing an analytic retrospective medical record study did not allow for studying such a correlation. Another limitation is that the sputum eosinophil count was not measured, but such a measurement is not routinely available in most centers and laboratories as it is time-consuming and needs special preparation.

## Conclusions

In conclusion, the FeNO, blood eosinophil count, IgE, and spirometric values correlated well with the severity of the eosinophilic airway inflammation in adult asthmatic patients. Additional prospective multi-center studies with multiple measurements of the biomarkers with a larger sample size are required to explore and verify the current results and ultimately support appropriate monitoring of asthma management.
